# Borrowed robes

**DOI:** 10.15252/embr.202154050

**Published:** 2021-10-28

**Authors:** Howy Jacobs

**Affiliations:** ^1^ Tampere University Tampere Finland

**Keywords:** Careers, Economics, Law & Politics, History & Philosophy of Science

## Abstract

Should scientists indulge their fantasies by writing fiction?

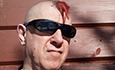

Like a substantial fraction of the literate population, I have a collection of unpublished novels in the drawer. Six of them in fact. Some of them were composed in barely more than a week, and others I have been struggling to complete for over 10 years: so maybe it is more accurate to say five and a half. Anyhow, most of them are good to go, give or take a bit of editorial redlining. Or, as my helpful EMBO editor would say, the removal of thousands of unnecessary adverbs and dubiously positioned commas.

What do I write about and why? My style is not unique but rather particular. I write fiction in the style of non‐fiction. My subject matter is somewhere in the general realms of science fiction, alternate history and political drama. Putting these ingredients together, and taking account of my purported day job as a serious scientist, it is easy to see why my fictional work is potentially subversive—which is one reason why I have been rather reluctant thus far to let it out of the drawer. At the very least, I should take pains to conceal my identity, lest it corrupts perceptions of my scientific work. Even if I regularly tell my students not to believe everything they read, it would impose far too great a burden on them if they came to question my peer‐reviewed articles purely on the basis of untrue statements published in my name, spoken by jaded politicians, washed‐up academics or over‐credulous journalists. Even if they are imaginary. Real journalists are theoretically bound by strict rules of conduct. But imaginary ones can do whatever they like.

Today, I noticed a passage in one of these unpublished works that is clearly written in the style of a young William Shakespeare, dealing with a subject matter that fits neatly into one of his most famous plays. In fact, the illusion was such that I was sure I must have lifted the passage from the play in question and set about searching for the quote, which I then could and should cite. Yet, all Internet searches failed to find any match. The character in whose mouth I placed the words was depicted as being in a delirious state where the boundaries of fact and fiction in his life were already blurred; borrowed identities being one of the themes of the entire novel and arguably of my entire oeuvre. But am I guilty here of plagiarism or poetry, in adopting the borrowed identity of my national playwright?

In another work, I lay great emphasis on the damaging role of mitochondrial reactive oxygen species (ROS) as the cause of biological ageing. I have even grafted this explanation onto a thinly disguised version of one of my most valued colleagues. Although there is some support for such a hypothesis from real science, including some papers that I have myself co‐authored, it is also a dangerously broad generalization that leads easily into wrong turnings and misconstructions—let alone questionable policies and diet advice. But, by advancing this misleading and overly simplistic idea in print, have I potentially damaged not only my own reputation, but that of other scientists whom I respect? Even if the author’s identity remains hidden.

In one novel, I fantasize that nuclear weapons, whilst they do undoubtedly exist, have in fact been engineered by their inventors so as never actually to work, thus preventing their possible misuse by vainglorious or lunatic politicians unconcerned with the loss of millions of lives and planetary ruin. But if any insane national leader—of which there are unfortunately far too many—would actually come to believe that my fiction in the style of non‐fiction were true, they might indeed risk the outbreak of nuclear war by starting a conventional one in order to secure their strategic goals.

Elsewhere, I vindicate one author of published claims that were manifestly based on falsified data, asserting him to have instead been the victim of a conspiracy launched to protect the family of an otherwise much respected American President. None of which is remotely true. Or at least there is no actual evidence supporting my ridiculous account.

I have great fun writing fiction of this kind. It is both liberating and relaxing to be able to ignore facts and the results of real experiments and just invent or distort them to suit an imaginary scenario. In an age when the media and real politicians have no qualms about propagating equally outrageous “alternative facts”, I can at least plead innocent by pointing out that my lies are deliberate and labelled as such, even if people might choose to believe them.

In a further twist, the blurb I have written to describe my latest work characterizes it as the “semi‐fictionalized” biography of a real person, who was, in fact, a distant relative of mine. But if it is semi‐fictionalized, which bits are true and which are made up? Maybe almost the whole thing is invented? Or maybe 99% of it is based on demonstrable facts? Maybe the subject himself concocted his own life story and somehow planted it in falsified documents and newspaper articles to give it an air of truth. Or maybe the assertion that the story is semi‐fictionalized is itself a fictional device, that is, a lie. Perhaps the central character never existed at all.

It is true (sic) that the most powerful fiction is grounded in fact—if something is plausible, it is all the more demanding of our attention. And, it can point the way to truths that are not revealed by a simple catalogue of factual information, such as in a scientific report.

But I have already said too much: if any of my novels ever do find their way into print, and should you chance to read them, I will be instantly unmasked. So maybe I’ll have to slot in something else in place of my pseudo‐Shakespearean verse, mitochondrial ROS hypothesis, defunct weapons of mass destruction and manipulated data manipulation.

